# 

**Published:** 1894-06

**Authors:** 


### TABLE OF CONTENTS.

ORIGINAL COMMUNICATIONS. PAGE

A Case of drawing the Lower Jaw Forward. By H. E. CUTTER, D.D.S., Cambridge, Mass. . . . . 353
Soft Foils and Plastic Stoppings. By DR. C. E. FRANCIS, New York . . . 356
Tin-Foil as a Filling-Material. By DR. BENJAMIN LORD, New York . . . 363
Diet: Its Relation to Tooth-Tissue. By L. ASHLEY FAUGHT, D.D.S. . . . 366
A New Form of Rubber Wedge. By WILLIAM HERBERT ROLLINS, Boston . 368

REPORTS OF SOCIETY MEETINGS.

New York Odontological Society . . . . 368
American Academy of Dental Science . . . . 380
Odontological Society of Pennsylvania . . . . 388
Harvard Odontological Society . . . . 403

EDITORIAL.

The One Discredited Material . . . . 406

BIBLIOGRAPHY . . . . 410

OBITUARY.

Resolutions of Respect to Dr. Eames . . . . 411

DOMESTIC CORRESPONDENCE.

Sponge Grafting . . . . 411

NOTES AND COMMENTS . . . . 412

CURRENT NEWS.

Pennsylvania State Dental Examining Board . . . . 414
Chicago Dental Society . . . . 414
Massachusetts Dental Society . . . . 414
First District Dental Society, State of New York . . . . 415
Connecticut Valley Dental Society . . . . 415

SELECTIONS.

The Dangers of Aconitine . . . . 415
Mercuric Chloride . . . . 416

